# Busulfan‐containing conditioning regimens in allogeneic hematopoietic stem cell transplantation for acute lymphoblastic leukemia: A Taiwan observational study

**DOI:** 10.1002/cnr2.1488

**Published:** 2021-06-30

**Authors:** Yu‐Hung Wang, Feng‐Ming Tien, Cheng‐Hong Tsai, Huai‐Hsuan Huang, Jia‐Hau Liu, Xiu‐Wen Liao, Jih‐Luh Tang, Ming Yao, Bor‐Sheng Ko

**Affiliations:** ^1^ Graduate Institute of Clinical Medicine, College of Medicine National Taiwan University Taipei Taiwan; ^2^ Division of Hematology, Department of Internal Medicine National Taiwan University Hospital Taipei Taiwan; ^3^ Department of Hematological Oncology National Taiwan University Cancer Centre Taipei Taiwan; ^4^ Tai‐Cheng Cell Therapy Centre National Taiwan University Taipei Taiwan

**Keywords:** acute lymphoblastic leukemia, allogeneic transplantation, busulfan, conditioning chemotherapy, Taiwan

## Abstract

**Background:**

Allogeneic stem cell transplantation (allo‐HSCT) is the ultimate cure for acute lymphoblastic leukemia (ALL).

**Aim:**

This study was performed to compare the outcomes of ALL patients receiving busulfan (Bu) with cyclophosphamide (Cy)‐based or total body irradiation (TBI)‐based regimen in a Chinese population.

**Methods:**

We enrolled 224 adult patients with ALL who received allo‐HSCT at National Taiwan University Hospital between 1997 and 2016.

**Results:**

The median age at transplantation was 33 years. Before allo‐HSCT, 75.9% of patients attained first or late complete remission. A total of 141 patients (62.9%) received Bu/Cy‐based conditioning, either myeloablative (MA) or reduced‐intensity stem cell transplantation (RIST), and 83 patients received a TBI‐based regimen (MA‐TBI). Patients receiving the MA‐Bu regimen had longer relapse‐free survival (RFS) than those receiving the MA‐TBI regimen (median, 24.1 vs. 6.7 months, *p* = .044). There was no difference in overall survival (OS, MA‐Bu vs. MA‐TBI vs. RIST‐Bu: 39.4 vs. 28.2 vs. 13.1 months, *p* = .276), treatment‐related mortality (TRM), or incidences of grade 3–4 acute graft‐versus‐host disease (GvHD). Among patients receiving identical GvHD prophylactic regimens, there was no difference between MA‐Bu and MA‐TBI groups regarding the incidence of grade 3–4 acute GvHD, grade 2–4, and all‐grade chronic GvHD. In subgroup analysis, patients receiving oral busulfan had comparable RFS and OS to the intravenous busulfan group (*p* = .436 and *p* = .236, respectively), but a higher TRM (25% vs. 9.8%, *p* = .016). In the multivariable analysis, disease status before allo‐HSCT was the only risk factor impacting RFS and OS.

**Conclusion:**

In summary, patients receiving Bu/Cy‐based or TBI‐based regimens as conditioning had similar results in terms of OS, TRM, and acute GvHD, whereas the use of myeloablative Bu/Cy resulted in a better RFS. A Bu‐based regimen could be an alternative conditioning choice for patients who are ineligible to receive TBI. Prospective and randomized controlled trials are warranted to validate the long‐term outcomes.

## 1 INTRODUCTION

Advances in the treatment of adult acute lymphoblastic leukemia (ALL) by small molecular agents and chimeric antigen receptor T‐cell therapy have greatly improved patient survival in the last decade.^1^, ^2^, ^3^, ^4^, ^5^, ^6^, ^7^ Meanwhile, recurrence is common in the post‐therapy course, which poses a challenge to long‐term remission.^8^, ^9^, ^10^, ^11^ Allogeneic hematopoietic stem cell transplantation (allo‐HSCT) is thus still considered the ultimate cure for ALL.^12^, ^13^, ^14^ An essential component of allo‐HSCT is the conditioning regimen, which eradicates the cancer cells and provides stem cell niches in the host bone marrow for the new stem cells. Total body irradiation (TBI) is effective against a variety of malignancies without sanctuary sites, such as the central nervous system, and therefore has been the gold standard conditioning regimen.^15^, ^16^ Complications of TBI include delayed growth and development in children, interstitial pneumonitis and secondary malignancies.^15^, ^17^, ^18^ TBI has been widely used in the Western world, while reports of its treatment effectiveness in the Chinese population have been rare. Busulfan (Bu) is an alkylating agent that has a potent effect on leukemia and can also serve as a common conditioning agent along with cyclophosphamide.^19^, ^20^ Nevertheless, the absorption of Bu in the gastrointestinal tract is quite variable among patients.^21^, ^22^ Some adverse effects, such as sinusoidal obstruction syndrome (SOS; previously known as veno‐occlusive disease [VOD]), restrict patients' and physicians' choices.^23^, ^24^, ^25^ Previous studies have confirmed the safety and efficacy of targeted‐dose Bu, which reduces patients' risks of SOS, treatment‐related mortality (TRM), and relapse.^26^, ^27^, ^28^, ^29^ The comparison of the treatment efficacies of TBI/cyclophosphamide (Cy) and busulfan (Bu)/Cy as conditioning regimens, both of which are major options for conditioning regimens for ALL, has long been unclear. Patients receiving different regimens might have moderately different outcomes from the perspective of relapse‐free survival (RFS), TRM, and the cumulative incidence of acute or chronic graft‐versus‐host disease (GvHD). However, no single regimen has clear benefits in terms of overall survival (OS).^30^, ^31^, ^32^, ^33^, ^34^ This real‐world observational study was performed to compare the outcomes of Chinese ALL patients receiving a Bu‐based or a TBI‐based regimen, and pre‐treatment parameters and therapy modalities were analyzed for risk stratification and survival analyses.

## 2 METHODS

### 2.1 Patients

We enrolled 224 ALL patients who received their 1st allo‐HSCT at National Taiwan University Hospital (NTUH) from January 1997 to December 2016. We retrospectively reviewed the medical records and obtained the clinical information. This study, along with the policy to waive informed consent, was approved by the Research Ethics Committee of NTUH (Project number: 201810058RIND).

### 2.2 Conditioning regimen before allogeneic hematopoietic stem cell transplantation

In this study, the conditioning regimens were categorized as follows: myeloablative TBI (MA‐TBI); MA‐Bu; and reduced intensity stem cell transplantation Bu (RIST‐Bu). The MA‐TBI protocol was administered as follows: TBI 150 centi‐gray (cGy) twice daily from Day −7 to Day −4 (total dose 1200 cGy) and cyclophosphamide IV 60 mg/kg/day on Day −3 and Day −2. The MA‐Bu regimen was administered as follows: busulfan IV 3.2 mg/kg/day or oral 4 mg/kg/day from Day −8 through Day −5 consecutively and cyclophosphamide IV 60 mg/kg/day on Day −3 and Day −2. RIST‐Bu protocol was administered as follows: fludarabine 30 mg/m^2^/day from Day −8 to Day −4 consecutively, busulfan IV 3.2 mg/kg/day or oral 4 mg/kg/day on Day −5 and Day −4, and cyclophosphamide IV 60 mg/kg/day on Day −2. Notably, the IV form of busulfan was introduced to our institute in 2009. The criteria used to use MA‐TBI, MA‐Bu or RIST‐Bu conditioning regimens are based on the patients' age, comorbidities, presence of high‐risk features (e.g., hyperleukocytosis, poor‐risk karyotypes, extramedullary disease, and disease status before transplant), and patients' willing after explanation of risks of adverse events of each regimen. The worth of mention is that the equipment for TBI had been in malfunction from August 2013 to August 2017; thus, patients who underwent HSCT during this period all received busulfan‐based conditioning regimens.

### 2.3 Prophylaxis of graft‐versus‐host disease

We used cyclosporin and methotrexate for GvHD prophylaxis in patients receiving MA‐TBI and MA‐Bu conditioning; cyclosporin and mycophenolate mofetil were used in the RIST‐Bu conditioning group. Rabbit anti‐thymoglobulin (ATG) was given to the patients who received Hematopoietic stem cells from the human leukocyte antigen (HLA)‐mismatched donors or the unrelated donor. A total dose of 5 mg/kg of body weight was given to the patients who received stem cells from the matched unrelated donors, and a total dose of 6 mg/kg of body weight was given to the patients who received stem cells from the haploidentical donors or mismatched unrelated donors. The ATG was divided into 2–3 days and given before the infusion of Hematopoietic stem cells.

### 2.4 Definitions

The transplant data, including demographics, underlying disease characteristics, transplantation procedures, and post‐HSCT complications, were collected according to the European Society for Blood and Marrow Transplantation Registry data collection forms and manuals (https://www.ebmt.org/registry/data-collection). The first infusion day of hematopoietic stem cells was defined as Day 0. OS was defined as the duration from Day 0 to the date of last follow‐up or death. RFS was the duration from Day 0 to the date of relapse, last follow‐up or death, whichever occurred first. TRM was defined as a death resulted from any cause other than relapse.

### 2.5 Statistical analysis

We used the Mann–Whitney *U* test to compare the medians of continuous variables with normal distributions. Fisher's exact test or the χ^2^ test were performed to examine the differences among discrete variables, including sex, responses, and recurrence in different treatment subgroups. Kaplan–Meier method was used to plot the survival curves and the log‐rank test was used to calculate the statistical significance. The Cox proportional hazards model was used in univariate and multivariable regression analyses. A *p* values less than .05 were considered statistically significant. All statistical analyses were performed with IBM SPSS Statistics 23 for Windows.

## 3 RESULTS

### 3.1 Patient characteristics

The patient characteristics are shown in Table 1. The median age at allo‐HSCT was 33 years, and 119 males and 105 females were included in our study. We stratified the adult ALL patients into three groups according to the types of conditioning chemotherapies (MA‐TBI, MA‐Bu, and RIST Bu; Table 1). Patients in MA‐TBI and MA‐Bu groups were younger than in those in the RIST‐Bu group (*p* < .001) and more male patients received myeloablative chemotherapy (MA‐TBI or MA‐Bu) rather than a reduced‐intensity regimen. There was no difference between the three groups in the distribution of WBC at diagnosis, presence of extramedullary disease, cytogenetic changes, disease status before allo‐HSCT, source of stem cell, and the dose of stem cells infusion. Although the majority of the patients received stem cells from the peripheral blood stem cell harvest, some patients in the MA‐TBI and MA‐Bu groups also received stem cells from bone marrow harvest (*p* < .001; Table 1). Moreover, in the MA‐Bu group, 46 (48.4%) patients received oral busulfan whereas only two (4.3%) patients in the RIST‐Bu group received oral busulfan (*p* < .001; Table 1).

**TABLE 1:** Clinical and laboratory features of patients receiving different conditioning regimens

Clinical characteristics	Total(*n* = 224)	MA‐TBI(*n* = 83)	MA‐Bu(*n* = 95)	RIST‐Bu(*n* = 46)	*p* value
Age^a^	33.3 (15.5–65.5)	28.1 (16.4–56.3)	30.9 (15.5–52.3)	51.8 (20.4–65.5)	<.001
Sex (*n*, %)					.001
Male	119 (53.1%)	56 (67.5%)	48 (50.5%)	15 (32.6%)	
Female	105 (46.9%)	27 (32.5%)	47 (49.5%)	31 (67.4%)	
Initial WBC (×10^3^/μl)^a^	23.6 (0.7–858.0)	30.0 (0.7–530.7)	22.6 (0.8–791.7)	15.5 (1.8–858.0)	.237
Extramedullary disease (*n*, %)	45 (20.1%)	18 (21.7%)	19 (20%)	8 (17.4%)	.843
Immunophenotype (*n*, %)					.461
B cell	137 (67.8%)	52 (70.3%)	53 (63.1%)	32 (72.7%)	
Pro‐B	18	8 (44.5%)	6 (33.3%)	4 (22.2%)	
Early Pre‐B	40	14 (35.0%)	15 (37.5%)	11 (27.5%)	
Pre‐B	34	13 (38.2%)	13 (38.2%)	8 (23.6%)	
Mature B	12	4 (33.3%)	5 (41.7%)	3 (25%)	
Unclassified B	33	13 (39.4%)	14 (42.4%)	6 (18.2)	
T cell	65 (32.2%)	22 (29.7%)	31 (36.9%)	12 (27.3%)	
Unknown	22	9	11	2	
Cytogenetics (*n*, %)					
*t*(9;22)	41 (22.7%)	13 (19.1%)	16 (22.2%)	12 (29.3%)	.468
Standard risk	101 (55.8%)	38 (55.9%)	41 (56.9%)	22 (53.7%)	.944
Poor risk, without *t*(9;22)	39 (21.5%)	17 (25.0%)	15 (20.8%)	7 (17.1%)	.611
Unknown	42	15	22	5	
Pre‐HSCT disease status (*n*, %)					.626
Relapse/refractory	54 (24.1%)	23 (27.7%)	21 (22.1%)	10 (21.7%)	
Complete remission	170 (75.9%)	60 (72.3%)	74 (77.9%)	36 (78.3%)	
CR1	114 (50.9%)	36 (60%)	55 (74.3%)	23 (63.9%)	
Late CR	56 (25.0)	24 (40%)	19 (25.7%)	13 (36.1%)	
Cell source (*n*, %)					<.001
BM	50 (22.3%)	28 (33.7%)	22 (23.2%)	0 (0%)	
PBSC	159 (71.0%)	54 (65.1%)	66 (69.5%)	39 (84.8%)	
BM + PBSC	15 (6.7%)	1 (1.2%)	7 (7.4%)	7 (15.2%)	
Donor (*n*, %)					.275
Sibling matched	123 (56.2%)	48 (60%)	53 (57%)	22 (47.8%)	
Relative, haplotype	17 (7.8%)	4 (5%)	6 (6.5%)	7 (15.2%)	
Unrelated donor	79 (36%)	28 (35%)	34 (36.5%)	17 (37.0%)	
Unknown	5				
Busulfan (*n*, %)					<.001
Oral			46 (48.4%)	2 (4.3%)	
Intravenous			49 (51.6%)	44 (95.7%)	
CD34+ cells (×10^6^/kg)^a^	4.81 (0.7–12.6)	4.75 (1.1–12.3)	4.79 (0.7–12.6)	4.69 (1.5–10.73)	.873

Abbreviations: BM, bone marrow; CR, complete remission; MA, myeloablative; PBSC, peripheral blood stem cell; RIST, reduced‐intensity stem cell transplantation; TBI, total body irradiation.

^a^ Median (range), at diagnosis.

### 3.2 Survival analysis

**FIGURE 1:**
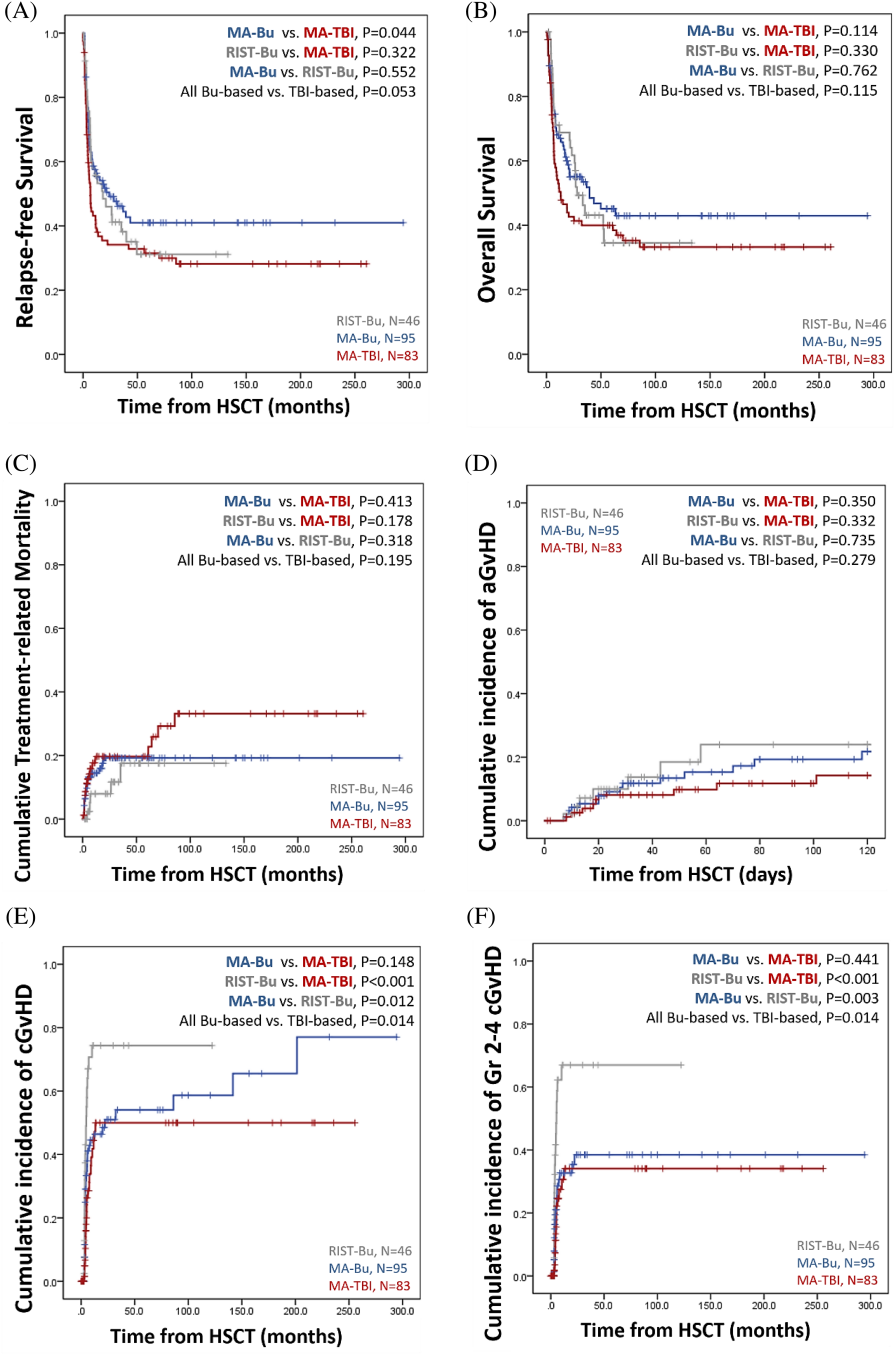
Kaplan–Meier plots of survival and cumulative incidence of graft‐versus‐host disease stratified by different conditioning regimens. (A) RFS, (B) OS, and (C) TRM of the 224 ALL patients receiving different conditioning regimens; cumulative incidence of (D) grade 3–4 acute GvHD at day+100, (E) all grade chronic GvHD, and (F) grade 2–4 chronic GvHD of the 224 ALL patients receiving different conditioning regimens

With a median follow‐up duration of 19.2 (range: 0.4–294.4) months, the RFS and OS of all patients were 11.7 months and 26.7 months, respectively. At the end of follow up, 32 (39%) patients in the MA‐TBI group, 48 (51%) patients in the MA‐Bu group, and 22 (48%) patients in the RIST‐Bu group remained alive. The relapse of disease was the main cause of death, accounting for more than 50% in all groups. In the MA‐Bu group, no patient died of GVHD while 13 (28%) patients succumbed to infection. In the subgroup analysis, the MA‐Bu group had significantly longer RFS than the TBI‐based group (median, 24.1 vs. 6.7 months, *p* = .044, Figure 1A). However, there was no significant difference in OS among patients receiving different conditioning regimens (median, MA‐Bu vs. MA‐TBI vs. RIST‐Bu: 39.4 vs. 28.2 vs. 13.1 months, *p* = .276, Figure 1B). By stratifying patients receiving busulfan‐based regimen on whether they were taking the oral or IV form, we found no differences in RFS and OS (median RFS, 8.5 vs. 26.3 months, *p* = .436; and median OS, 17 vs. 39.4 months, *p* = .236, respectively, Figure S1A,B). Besides, since the recruitment of patients spanned a long period, we further analyzed patients receiving busulfan orally or intravenously transplanted in different eras to exam possible chronologic effect. Patients were separated by the median calendar year of transplantation, in 2004 for patients receiving busulfan orally and in 2014 for patients receiving busulfan intravenously, respectively. The survival of patients receiving transplantation more contemporarily did not overwhelm their counterparts (Figure S2), notwithstanding that this should be interpreted cautiously given that there was discrepancy of post‐transplant follow‐up period and the bi‐modal distribution.

### 3.3 Treatment‐related mortality

There was no difference in cumulative TRM (MA‐Bu vs. MA‐TBI vs. RIST‐Bu: 17% vs. 21.7% vs. 10.9%, *p* = .308, Figure 1C) among all regimen groups. However, patients who took oral busulfan had a higher risk of TRM than those who received IV busulfan (25% vs. 9.8%, *p* = .016, Figure S1C).

### 3.4 Graft‐versus‐host disease

The cumulative incidences of grade 3–4 acute GvHD at day +100 were not significantly different among the three groups (MA‐Bu vs. MA‐TBI vs. RIST‐Bu: 15.8% vs. 10.8% vs. 15.2%, *p* = .445, Figure 1D), whereas the cumulative incidence of all‐grade chronic GvHD in the RIST‐Bu group was significantly higher than those in the other two groups (MA‐Bu vs. MA‐TBI vs. RIST‐Bu: 41.1% vs. 27.7% vs. 56.5%, *p* = .001, Figure 1E). The majority (80.8%) of chronic GvHD in the RIST and Bu‐based groups were grade 2–4, and the incidence of grade 2–4 chronic GvHD was consistently higher than those in the other two groups (MA‐Bu vs. MA‐TBI vs. RIST‐Bu: 24.2% vs. 18.1% vs. 45.7%, *p* < .001, Figure 1F). We next performed subgroup analysis to dissect the potential impact of different prophylactic measures. Interestingly, in MA‐Bu and RIST‐Bu groups, patients receiving ATG prophylaxis (and HSCs from nonsibling‐matched donors) had a higher incidence of grade 3–4 acute GvHD at day +100 than their counter partners (Figure S3A,B, *p* = .073 and *p* = .005, respectively) while there was no such difference in the MA‐TBI group (Figure S3C, *p* = .802). On the other hand, among patients receiving identical prophylactic regimens, there was no difference between MA‐Bu and MA‐TBI groups regarding the incidence of grade 3–4 acute GvHD, grade 2–4, and all‐grade chronic GvHD (Figure S4A–F).

### 3.5 Extramedullary disease

**FIGURE 2:**
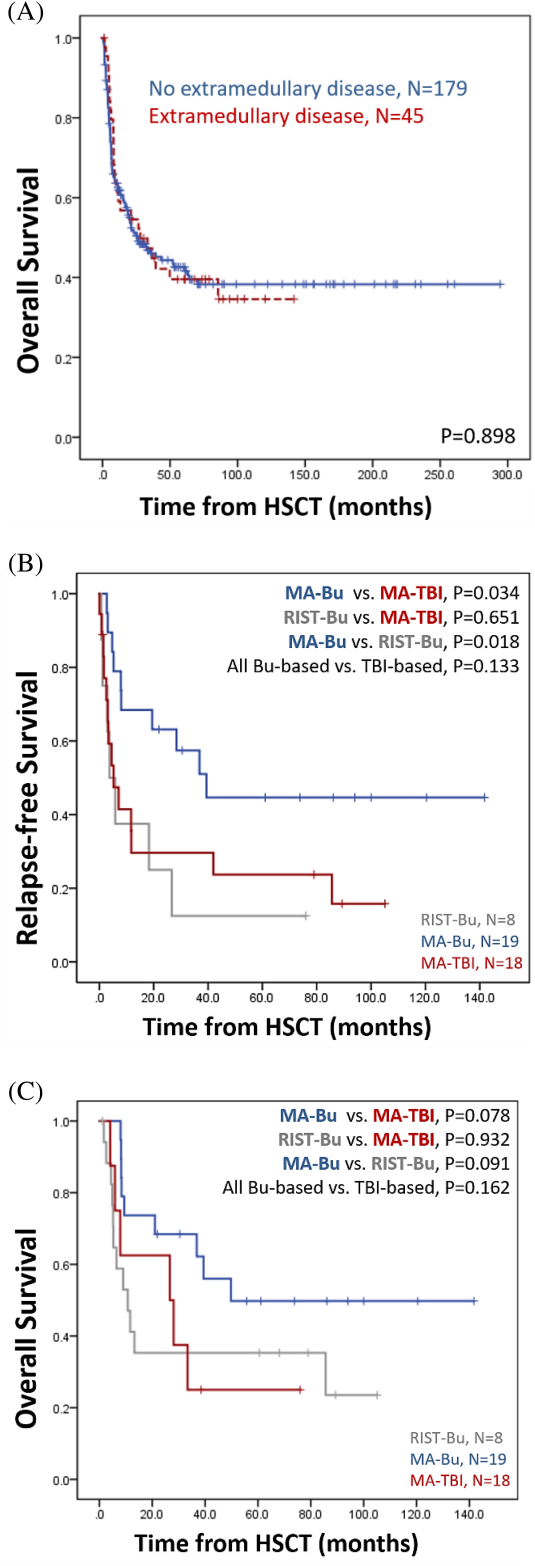
Kaplan–Meier plots in extramedullary disease subgroup analysis. (A) OS of the 224 ALL patients stratified by extramedullary disease. (B) RFS and (C) OS of the 45 ALL patients with extramedullary disease receiving different conditioning regimens

In this cohort, patients with extramedullary disease at diagnosis did not have significantly shorter OS than those without (median, with extramedullary disease vs. without, 28.1 vs. 25.8 months, *p* = .898, Figure 2A). This might imply that allo‐HSCT can overcome the expected poor prognosis of extramedullary disease. The MA‐Bu group outperformed the other two regimens in regard to RFS (median, MA‐Bu vs. RIST‐Bu, 39.4 vs. 3.8 months, *p* = .018; MA‐Bu vs. MA‐TBI, 39.4 vs. 5.2 months, *p* = .034, Figure 2B) and OS (median, MA‐Bu vs. RIST‐Bu, 109.9 vs. 51.2 months, *p* = .091; MA‐Bu vs. MA‐TBI, 109.9 vs. 60.4 months, *p* = .078, Figure 2C).

### 3.6 Multivariable analysis

For multivariable analysis, we included parameters with a *p* value <.15 in univariate Cox regression analysis (Table 2) and biologically relevant parameters as covariates, including age, WBC count at diagnosis, karyotype, presence of extramedullary disease, disease status before HSCT, and the conditioning regimen. In multivariable analysis, disease status before HSCT and MA‐Bu conditioning were factors that affected RFS, while disease status before HSCT was the only risk factor for OS. In the subgroup analysis of patients with extramedullary diseases, MA‐Bu conditioning was found to have protective effects compared to TBI‐based conditioning, while status before transplant remained an independent risk factor for OS (Table 3).

**TABLE 2:** Univariate and multivariable analyses of RFS and OS of the 224 patients with acute lymphoblastic leukemia

Variable	Univariate			Multivariate		
	HR	95% CI	*p* value	HR	95% CI	*p* value
**RFS**						
Age (≥ vs. < 40 y/o)	0.93	0.64–1.36	.710	1.21	0.68–2.14	.513
Initial WBC (≥10 vs. < 10K/μl)	1.13	0.76–1.68	.548	1.29	0.78–2.15	.314
Cytogenetics (Standard vs. High risk^a^)	0.94	0.64–1.38	.750	1.10	0.70–1.72	.678
Extramedullary (Yes vs. No)	1.16	0.78–1.74	.460	1.23	0.74–2.05	.420
Immunophenotype (T vs. B)	1.18	0.82–1.71	.368			
Pre‐HSCT disease status (vs. CR1)						
Late CR	1.61	1.07–2.42	.023	1.67	1.01–2.75	.047
Relapse/refractory	2.86	1.91–4.27	<.001	2.61	1.51–4.49	.001
Cell source (PBSC vs. BM/BM + PBSC)	1.15	0.79–1.67	.462			
Donor (Unrelated donor vs. Sibling)	1.01	0.90–1.14	.816			
Conditioning (vs. MA, TBI‐based)						
MA‐Bu based^f^	0.68	0.47–0.99	.046	0.61	0.38–0.97	.035
RIST‐Bu based^f^	0.79	0.51–1.24	.304	0.65	0.34–1.23	.186
Busulfan (intravenous vs. oral)	0.84	0.53–1.32	.437			
CD34+ cells (×10^6^)	0.99	0.91–1.08	.876			
**OS**						
Age (≥ vs. < 40 y/o)	0.98	0.66–1.46	.927	1.41	0.77–2.56	.263
Initial WBC(≥10 vs. < 10K/μl)	0.99	0.66–1.49	.979	1.21	0.71–2.05	.490
Cytogenetics (Standard vs. High risk^a^)	0.91	0.61–1.37	.665	1.09	0.68–1.75	.719
Extramedullary (Yes vs. No)	1.46	0.84–2.55	.184	1.08	0.63–1.84	.792
Immunophenotype (T vs. B)	1.13	0.77–1.67	.529			
Pre‐HSCT disease status						
Late CR vs. CR1	1.77	1.16–2.72	.009	1.83	1.08–3.10	.024
Relapse/refractory vs. CR1	2.85	1.87–4.34	<.001	2.77	1.56–4.93	.001
Cell source (PBSC vs. BM/BM + PBSC)	1.06	0.72–1.55	.782			
Donor (Unrelated donor vs. Sibling)	1.05	0.92–1.18	.491			
Conditioning						
MA‐Bu based vs. MA, TBI‐based	0.73	0.49–1.09	.120	0.65	0.39–1.06	.083
RIST‐Bu based vs. MA, TBI‐based	0.79	0.49–1.27	.331	0.62	0.31–1.24	.176
Busulfan (intravenous vs. oral)	0.75	0.47–1.21	.238			
CD34+ cells (×10^6^)	0.99	0.92–1.09	.970			

*Note*: Statistically significant if *p* < .05.

Abbreviations: BM, bone marrow; CI, confidence interval; CR, complete remission; HSCT, hematopoietic stem cell transplantation; HR, hazard ratios; MA, myeloablative; PBSC, peripheral blood stem cell; RIST, reduced‐intensity stem cell transplantation; TBI, total body irradiation.

^a^ High risk: *t*(9;22)/*BCR‐ABL1*, t(v;11q23)/*KMT2A (MLL)* rearrangements, or hypodiploidy (<44 chromosomes).

**TABLE 3:** Multivariable analysis for RFS and OS of the 45 ALL patients with extramedullary diseases

Variable	RFS			OS		
	HR	95%CI	*p*	HR	95%CI	*p*
Age (≥ vs. < 40 y/o)	0.74	0.19–2.82	.663	0.66	0.17–2.55	.547
Initial WBC (≥10 vs. < 10K/μl)	0.51	0.14–1.90	.312	0.54	0.14–2.06	.365
Cytogenetics (Standard vs. High risk^a^)	3.93	1.37–11.23	.011	2.49	0.80–7.73	.115
Pre‐HSCT disease status						
Late CR vs. CR1	2.07	0.64–6.72	.226	4.32	1.31–14.27	.016
Relapse/refractory vs. CR1	12.27	1.90–79.12	.008	20.88	3.04–143.3	.002
Conditioning						
MA‐Bu based vs. MA, TBI‐based	0.37	0.12–1.16	.088	0.56	0.16–1.98	.369
RIST‐Bu based vs. MA, TBI‐based	0.93	0.24–3.62	.919	1.63	0.42–6.28	.478

*Note*: Statistically significant if *p* < .05.

Abbreviations: CI, confidence interval; CR, complete remission; HSCT, hematopoietic stem cell transplantation; HR, hazard ratios; MA, myeloablative; RIST, reduced‐intensity stem cell transplantation; TBI, total body irradiation.

^a^ High risk: *t*(9;22)/*BCR‐ABL1*, *t*(v;11q23)/*KMT2A (MLL)* rearrangements, or hypodiploidy (<44 chromosomes).

## 4 DISCUSSION

This is the first study comparing transplant outcomes in Chinese ALL patients receiving TBI‐ and Bu‐based conditioning therapy to the extent of our knowledge. A higher proportion of patients (63.4%) in our study received Bu‐based conditioning compared with patients in Western cohorts (4.2%–52.6%, Table 4).^30^, ^31^, ^32^, ^33^, ^34^, ^35^, ^36^, ^37^, ^38^ Long‐term TBI toxicities, including delayed growth and secondary malignancy, might be the main reason preventing our patients from receiving irradiation therapy. Additionally, we explored patient outcomes based on the form of busulfan administered (IV or oral form). Patients receiving IV busulfan had comparable RFS and OS to the oral group and a lower TRM rate, which could result from more stable pharmacokinetics; however, drug level monitoring was not routinely performed in our institute. This cohort could provide some directive for the real‐world practice where TBI or therapeutic drug monitoring of Bu are not available.

**TABLE 4:** Selected studies comparing the outcomes of patients with acute lymphoblastic leukemia receiving busulfan‐based or total body irradiation‐based conditioning regimens

Cohort	Patient number	OS	RFS/DFS/PFS	NRM/TRM	RI	aGVHD (II‐IV)	cGVHD	Summary/notes
Mitsuhashi, 2016^a^	TBI = 2028	59%	RFS 55.0%	NRM 20.8%	22.8%	40.4%	37.6% at 2 yr	TBI over PO BU for OS
	PO BU = 60	48.2%	RFS 44.6%	NRM 26.1%	28.5%	36.8%	31.5% at 2 yr	
	IV BU = 42	37.4%	RFS 34.2%	NRM 27.0%	32.6%	33.3%	40.1% at 2 yr	
Kebriaei, 2018^a^	TBI = 819	49%	DFS 45%	TRM 27%	29%	40.0%	55% at 3 yr	Similar OS and DFS
	BU = 299	46%	DFS 37%	TRM 22%	42%	47.0%	49% at 3 yr	
Candoni, 2019^b^	TBI = 221	HR: 0.86, 95% CI:	PFS HR: 0.72, 95% CI: 0.55–0.94	NRM HR: 1.16, 95% CI:	HR: 0.56, 95% CI:	Not reported	HR: 1.45, 95% CI: 0.93–2.26	Favor TBI for PFS and RI
	CT = 220	0.65–1.14		0.78–1.72	0.39–0.79			
Eroglu, 2013^c^	TBI = 45	53.2%	EFS 27.2%	TRM 9.0%	51%	22.2%	31.1%	Favor TBI for OS, EFS and RI
	BU = 50	30.9%	EFS 18.8%	TRM 16.0%	76%	26.0%	24.0%	
Granados, 2000^c^ ^,^ ^d^	TBI = 114	Not reported	EFS 43%	TRM 17% at 18 mo	47%	30.3%	7.9% extensive	Favor TBI for EFS and RI
	BU = 42		EFS 22%	TRM 22% at 18 mo	71%	23.8%	0% extensive	
Sakellari, 2018^a^	TBI = 84	46.7%; 57.6%^e^	DFS 46.1%	TRM 27.7%	Not reported	Not reported	48%^e^	Favor TBI in patients younger than 40 years
	BU = 67	35.8%; 39.7%^e^	DFS 35.4%	TRM 24.1%			27.4%	
Giebel, 2017	TBI = 504	69.5%	LFS 61.6%	NRM 17.3%	21.1%	Not reported	Not reported	Favor TBI for LFS and RI
	CT = 58	64.0%	LFS 49.7%	NRM 17.6%	32.7%			
Nishiwaki, 2016^f^ ^,^ ^g^	TBI = 310	HR 1.3, 95% CI: 0.65–2.57	LFS HR: 1.42, 95% CI: 0.79–2.57	NRM HR 1.51, 95% CI: 0.64–	HR 1.18, 95% CI:	Not reported	Not reported	Similar outcomes in patients under age of 55
	CT = 14			3.52	0.51–2.72			
Abdelaty, 2020	TBI = 78	42% at 2 yr	DFS 80% at 2 yr	NRM 38.5%	11.5%	33.3%^h^	30.8%	Favor TBI for EFS and RI; no significant difference in OS, DFS, and NRM
	BU = 41	44% at 2 yr	DFS 55% at 2 yr	NRM 48.8%	26.8%	36.6%	17.1%	
NTUH (this study)	TBI = 83	28.2 mo	RFS 6.7 mo	TRM 21.7%	44.6%	10.8%^h^	27.7%	Favor MA, BU‐based group for RFS
	MA, BU = 95	39.4 mo	RFS 24.1 mo	TRM 17.0%	33.7%	15.8%	41.1%	
	RIC, BU = 46	13.1 mo	RFS 18.3 mo	TRM 10.9%	47.8%	15.2%	56.5%	

Abbreviations: BU: busulfan‐based regimen; CI, confidence interval; CT: chemotherapy; IV: intravenous form; PO: oral form; RI, relapse incidence; TBI: total body irradiation.

^a^ Followed up at 5 years post‐transplant.

^b^ TBI‐based vs. Bu‐based, Bu as reference.

^c^ Follow‐up at 3 years post‐transplant.

^d^ Ninety patients received allogenic transplant, and 66 patients received autologous transplant.

^e^ In patients younger than 40 years.

^f^ Bu‐based vs. TBI‐based, TBI as reference.

^g^ Subanalyses of 324 patients under the age of 55 within the whole cohort treated with a myeloablative conditioning regimen.

^h^ All grades included.

While there is currently no firm consensus on the best conditioning therapy for allo‐SCT in adult patients with ALL, the results from previous studies provided evidence in modest favor of TBI (Table 4). Kebriaei et al. analyzed data from the Center for International Blood and Marrow Transplant Research (CIBMTR), revealing that patients using Bu had lower TRM (Bu 19% vs. TBI 25%, *p* = .04) but a higher relapse rate (Bu 37% vs. TBI 28%, *p* = .007) than patients using TBI.^33^ Compared with TBI‐based conditioning, Bu‐based conditioning led to similar disease‐free survival (DFS) and OS following allo‐SCT for ALL. Meanwhile, Mitsuhashi and colleagues conducted an analysis to compare TBI/Cy, oral Bu/Cy, and IV Bu/Cy in a cohort of 2130 Japanese patients, most of whom received TBI/Cy. The oral Bu/Cy group had a shorter OS than the TBI/Cy group, while the IV Bu/Cy group had comparable OS to the TBI/Cy group.^34^ No between‐group differences were seen in the incidence of non‐relapse mortality (NRM), relapse, acute GvHD, or chronic GvHD.

Herein, we present our transplant experience with ALL patients in Taiwan. The survival outcome of our cohort is comparable to those in other recently published studies (Table 4). The TRM of our patients seemed to be acceptable, yet the incidence of relapse (33.7%–47.8%) remained alarming. While the studies by Kebriaei et al. and Abdelaty et al. revealed a markedly increased relapse incidence in the busulfan group,^33^, ^38^ the relapse rates among three groups in our study were not that different. Interestingly, in a recent study, Speziali and colleagues analyzed outcomes of 146 ALL patients receiving TBI/Cy (1200 cGy) or fludarabine, busulfan, and low‐dose TBI (400 cGy) as conditioning regimens. The Flu/Bu/TBI group had a significantly lower incidence of relapse than the TBI/Cy group (18.5% vs. 31.5% at 2‐year, *p* = .05), while there was no difference in OS, PFS, and NRM, implicating an alternative combination of low‐dose TBI and Bu.^39^

Regarding survival, our patients in the MA‐Bu group had a better RFS than those in the MA‐TBI group. Nevertheless, the survival benefit of RFS conferred by Bu was not extended to long‐term OS. One explanation might be higher mortality rates resulted from late relapse and infection (Table 5). The overall relapse incidence was higher in the TBI group (44.6%) than in the MA‐Bu group (33.7%), but the MA‐Bu group caught up in terms of relapse‐related mortality. The MA‐Bu group had a higher rate (1.76‐fold) of death due to infection. Improvements in the management of disease relapse and infection after transplant might particularly help improve patient survival. In patients with extramedullary diseases, the MA‐Bu group consistently had longer RFS than the other two groups. There was also a trend towards longer OS, lower relapse incidence and TRM in the MA‐Bu group. This could be contrary to our impression of the anti‐leukemic effect of TBI on the sanctuary sites. As intrathecal chemotherapy was routinely performed for adult ALL patients in our institute, this could remunerate the suboptimal penetration of busulfan into the central nervous system. Another possible confounding factor is the timing of the transplant. More patients with extramedullary disease in the MA‐Bu group received allo‐SCT in their first complete remission (CR, MA‐Bu vs. MA‐TBI: 94.7% vs 66.7%, *p* = .042) rather than in latent CR. Excluding patients in the RIST‐Bu group and those not in remission, the difference in RFS and OS would become trivial (*p* = .97 and *p* = .841, respectively). Furthermore, the rate of TRM was higher in the MA‐TBI group than in the MA‐Bu group (22.2% vs 3.7%). Lastly, the dose of TBI (12 Gy) is inferior to higher doses (≥13 Gy), which was promoted by Marks et al.^16^ For patients receiving allo‐SCT, not in first CR, the risks of relapse and mortality might be diminished with TBI doses >13 Gy.

**TABLE 5:** Causes of death by treatment group

Clinical Characteristics	MA‐TBIDeath (*n* = 51)	MA‐BuDeath (*n* = 47)	RIST‐BuDeath (*n* = 24)	*p* value
Relapse	29 (56.9%)	28 (59.6%)	17 (70.8%)	.504
Graft failure	0	1 (2.1%)	0	.447
GVHD	6 (11.8%)	0	4 (16.7%)	.025*
Infection	8 (15.7%)	13 (27.7%)	1 (4.2%)	.044*
Interstitial pneumonitis or ARDS	3 (5.9%)	1 (2.1%)	0	.350
Secondary malignancy	1 (2.1%)	2 (4.3%)	1 (4.2)	.786
Other	2 (3.9%)	0	0	.243
Unknown	2 (3.9%)	2 (4.3%)	1 (4.2%)	.996

*Note*: *Indicating statisticallysignificant with *P* < 0.05.

Abbreviations: ARDS, acute respiratory distress syndrome; GVHD, graft‐versus‐host disease.

There are several limitations to our study. First, its retrospective nature imposed diverse sources of biases and temporal confounding factors that were difficult to assess. Although prospective and randomized trials for allogenic transplantation in ALL are challenging in clinical practice due to the complexity of the disease nature and treatment course, they are warranted and will be appreciated. Second, targeted drug monitoring was not routinely implemented in our institute. Regardless of the lack of pharmacokinetic data in patients receiving oral Bu, this subgroup had a higher TRM than those receiving IV busulfan, which was consistent with previous studies that showed that a more stable Bu pharmacokinetic level with IV dosing and the reductions in toxicities. Moreover, some bias may be recondite since the recruitment of patients spanned a long period. Although we confirmed that the survival of patients receiving transplantation more contemporarily did not overwhelm their counterparts, potential underlying confounding factors could not be all excluded. Despite these limitations, multivariable analysis in our study confirmed that there were no significant differences between the Bu‐based and TBI‐based groups in terms of OS, and MA‐Bu conditioning might improve RFS in eligible patients. These results imply that the Bu‐based regimen might improve patient outcomes in adult patients with ALL by reducing treatment toxicity and mortality. In the meantime, strategies for the prevention and salvage of disease relapse, which accounted for more than 50% of the deaths, should also be further investigated and improved.

In summary, this study provided a risk stratification and survival analysis of ALL patients undergoing allo‐SCT and demonstrated that a Bu‐based regimen could be an alternative conditioning choice for patients who are ineligible to receive TBI. Larger‐scale, prospective and randomized controlled trials are challenging but warranted to compare and validate the long‐term outcome of patients receiving Bu‐based and TBI‐based conditioning before transplant.

## CONFLICT OF INTEREST

The authors are not aware of any affiliations, memberships, funding, or financial holdings that might be perceived as affecting the objectivity of this study.

## AUTHOR CONTRIBUTIONS

**Yu‐Hung Wang:** Formal analysis; visualization; writing‐original draft. **Feng‐Ming Tien:** Data curation; resources. **ChengHong Tsai:** Data curation; resources. **Huai‐Hsuan Huang:** Data curation; resources. **Jia‐Hau Liu:** Data curation; resources. **Xiu‐Wen Liao:** Data curation; project administration; resources. **Jih‐Luh Tang:** Data curation; project administration; resources. **Ming Yao:** Data curation; project administration; resources. **Bor‐Sheng Ko:** Conceptualization; funding acquisition; investigation; resources; supervision; writing‐original draft.

## ETHICS STATEMENT

This study, along with the policy to waive informed consent, was approved by the Research Ethics Committee of NTUH (Project number: 201810058RIND). This article does not use any samples from human or animal subjects.

## Supporting information


**Figure S1.** Kaplan‐Meier plots of survival stratified by different forms of busulfan (a) RFS, (b) OS, and (c) TRM of the 141 ALL patients receiving different forms of busulfan‐based conditioning regimens.
**Figure S2.** Kaplan‐Meier plots of survival stratified by different forms of busulfan and calendar year of transplantation. RFS (a) and OS (b) of 48 patients receiving busulfan orally before and after 2004; and RFS (c) and OS (d) of 93 patients receiving busulfan orally before and after 2014.
**Figure S3.** Kaplan‐Meier plots of s cumulative incidence of graft‐vs.‐host disease (GvHD) stratified by different conditioning regimens and GvHD prophylactic regimens. In MA‐Bu (a) and RIST‐Bu (b) groups, patients receiving ATG prophylaxis (and HSCs from non‐sibling‐matched donors) had a higher incidence of grade 3–4 acute GvHD than their counter partners while there was no such difference in the MA‐TBI group (c).
**Figure S4.** Cumulative incidence of graft‐vs.‐host disease (GvHD) stratified by different conditioning regimens (MA‐Bu vs. MA‐TBI) and GvHD prophylactic regimens. There was no difference between MA‐Bu and MA‐TBI groups regarding the incidence of grade 3–4 acute GvHD (a and b), grade 2–4 (c and d), and all‐grade chronic GvHD (e and f).

## Data Availability

The datasets generated during and/or analyzed during the current study are available from the corresponding author on reasonable request.
