# Supplementary material for: Busulfan‐containing conditioning regimens in allogeneic hematopoietic stem cell transplantation for acute lymphoblastic leukemia: A Taiwan observational study
Source: Cancer Rep (Hoboken). 2021 Jun 30;5(3):e1488. doi: 10.1002/cnr2.1488 (PMC8955073; doi:10.1002/cnr2.1488)

Supplement Figure 1. Kaplan-Meier plots of survival stratified by different forms of busulfan

(a) RFS, (b) OS, and (c) TRM of the 141 ALL patients receiving different forms of busulfan-based conditioning regimens.

a

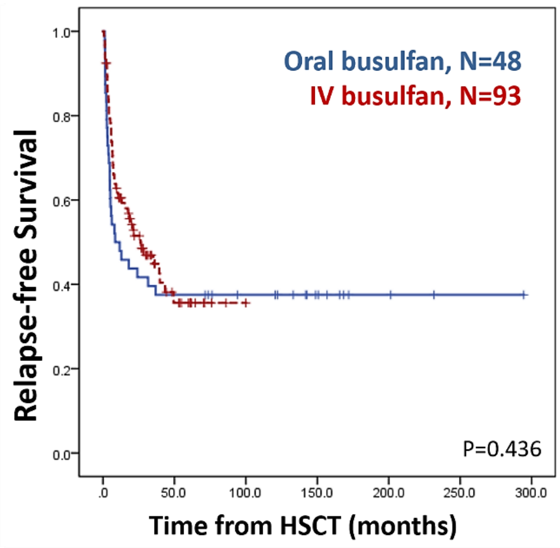

b

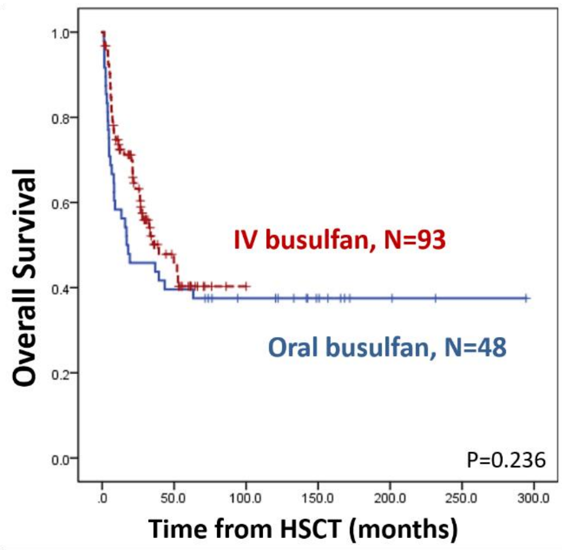

c

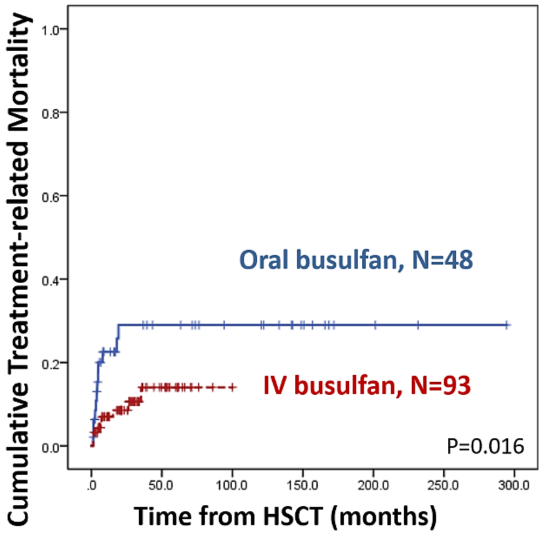

Supplement Figure 2. Kaplan-Meier plots of survival stratified by different forms of busulfan and calendar year of transplantation. RFS (a) and OS (b) of 48 patients receiving busulfan orally before and after 2004; and RFS (c) and OS (d) of 93 patients receiving busulfan orally before and after 2014.

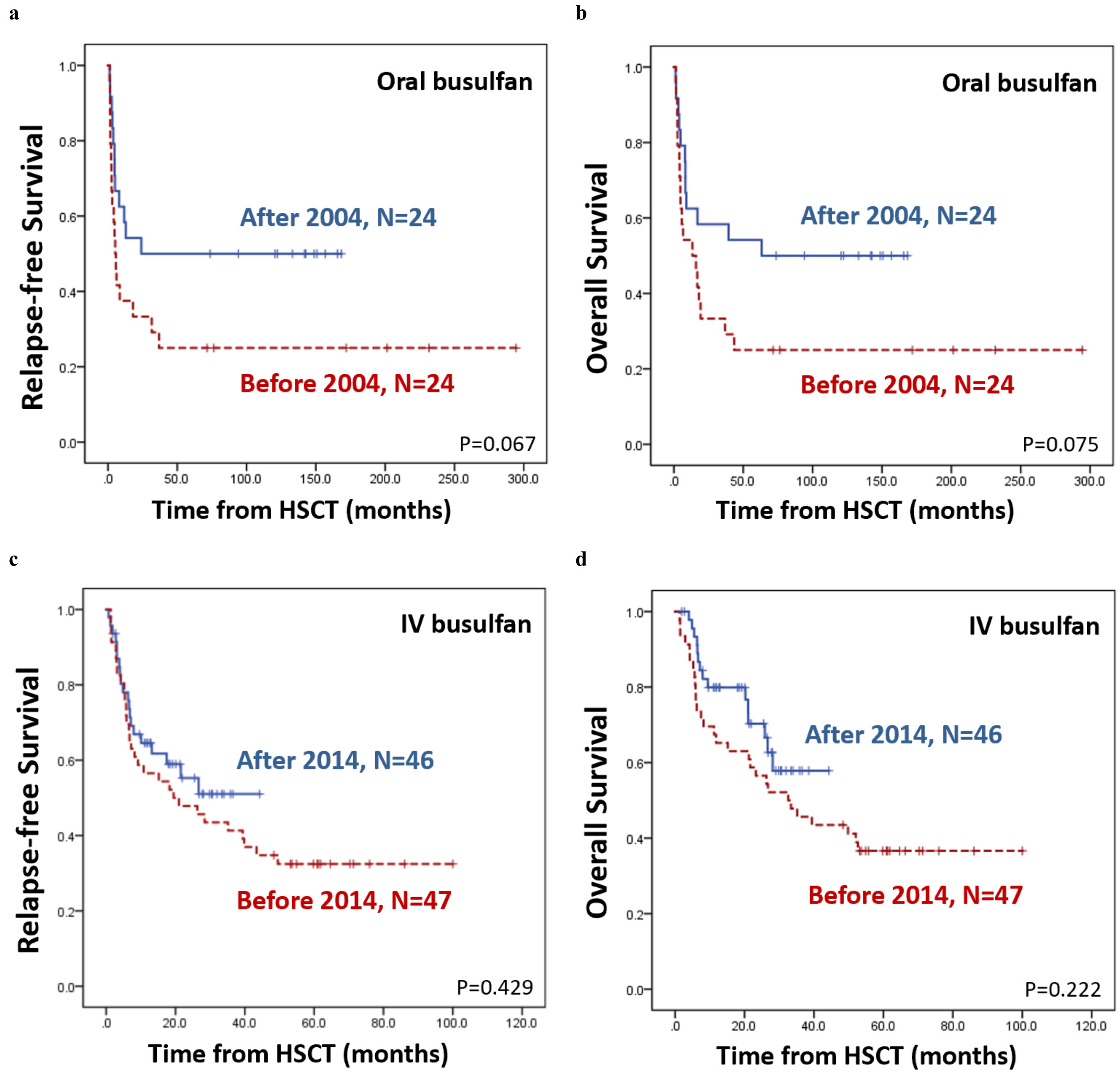

Supplement Figure 3. Kaplan-Meier plots of s cumulative incidence of graft-vs.-host disease (GvHD) stratified by different conditioning regimens and GvHD prophylactic regimens. In MA-Bu (a) and RIST-Bu (b) groups, patients receiving ATG prophylaxis (and HSCs from non-sibling-matched donors) had a higher incidence of grade 3-4 acute GvHD than their counter partners while there was no such difference in the MA-TBI group (c).

a

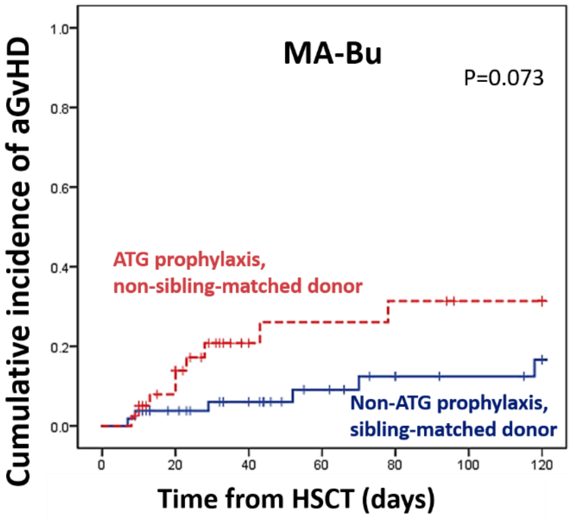

b

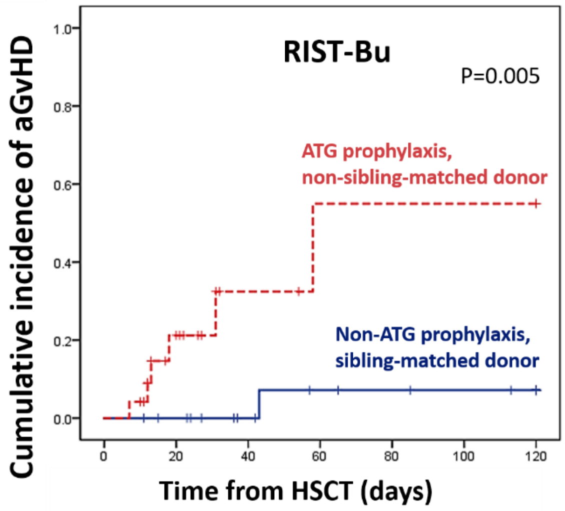

c

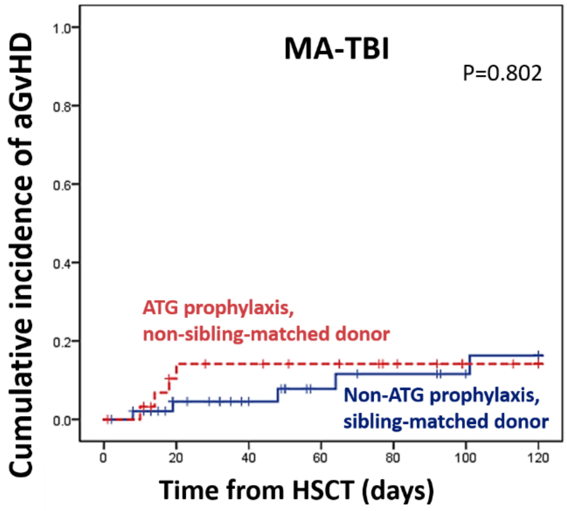

Supplement Figure 4. Cumulative incidence of graft-vs.-host disease (GvHD) stratified by different conditioning regimens (MA-Bu *vs.* MA-TBI) and GvHD prophylactic regimens. There was no difference between MA-Bu and MA-TBI groups regarding the incidence of grade 3-4 acute GvHD (a and b), grade 2-4 (c and d), and all-grade chronic GvHD (e and f).

a

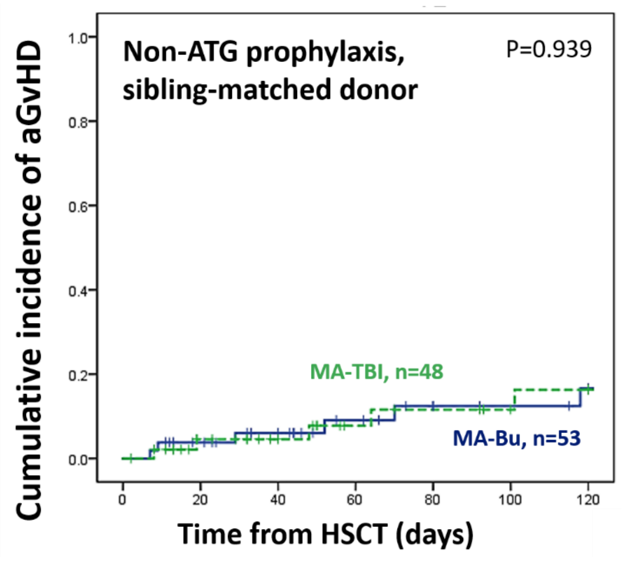

b

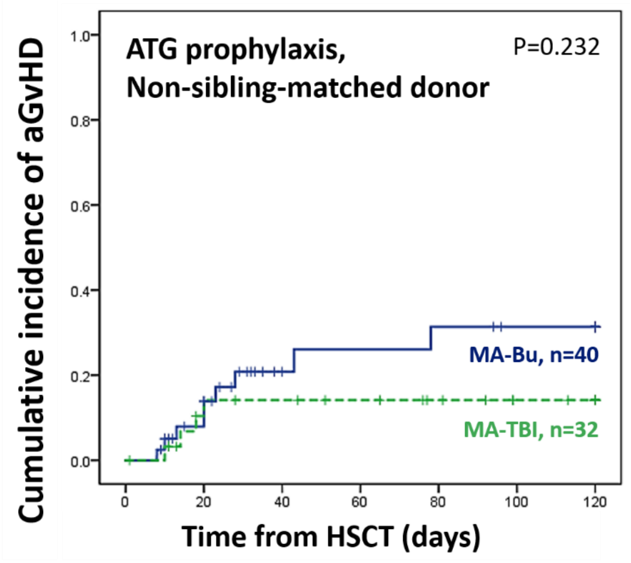

c

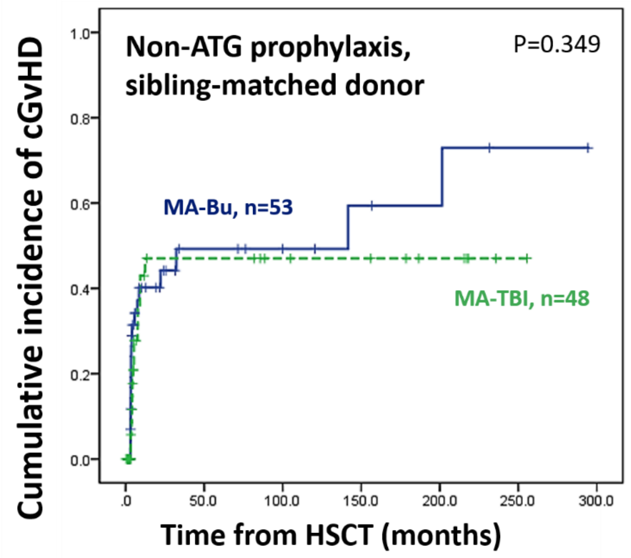

d

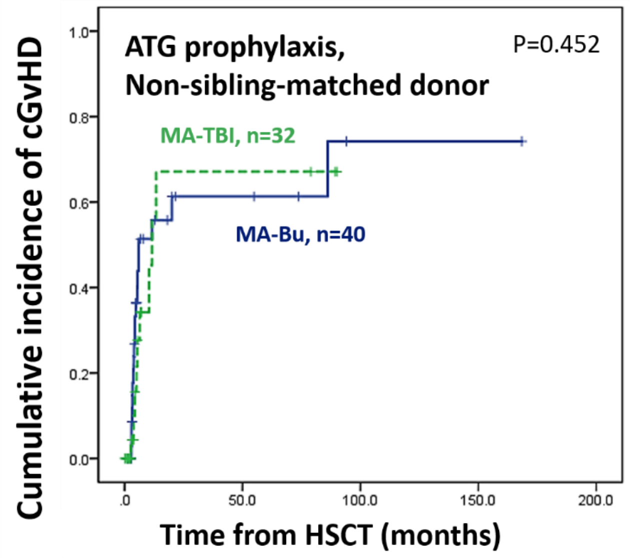

e

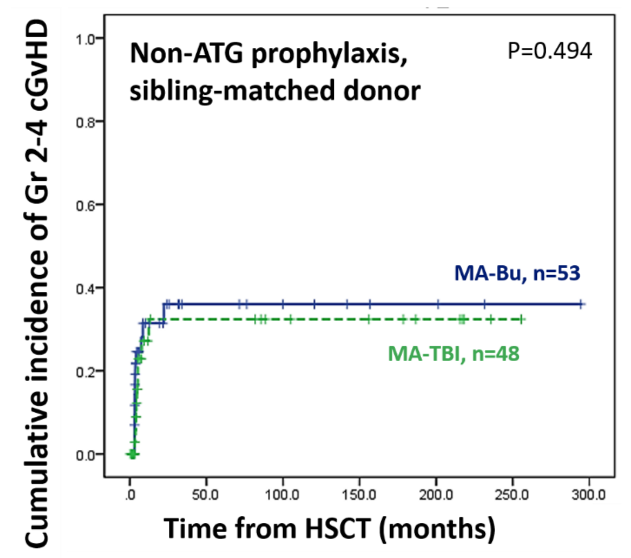

f

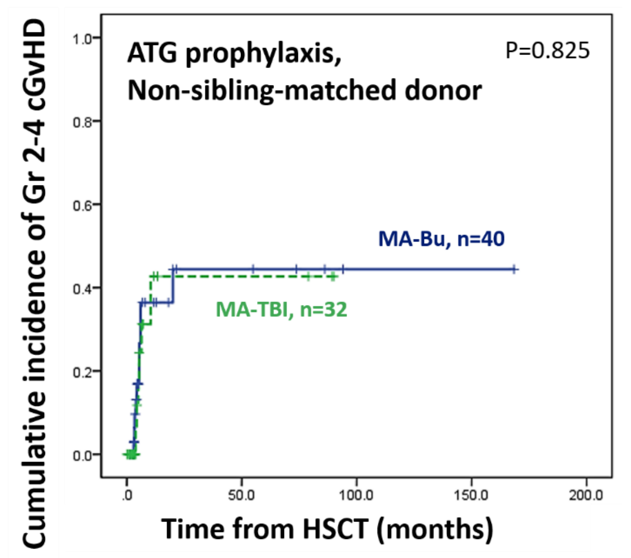

Supplement: Supplementary file 1 — Figure S1. Kaplan‐Meier plots of survival stratified by different forms of busulfan (a) RFS, (b) OS, and (c) TRM of the 141 ALL patients receiving different forms of busulfan‐based conditioning regimens. Figure S2. Kaplan‐Meier plots of survival stratified by different forms of busulfan and calendar year of transplantation. RFS (a) and OS (b) of 48 patients receiving busulfan orally before and after 2004; and RFS (c) and OS (d) of 93 patients receiving busulfan orally before and after 2014. Figure S3. Kaplan‐Meier plots of s cumulative incidence of graft‐vs.‐host disease (GvHD) stratified by different conditioning regimens and GvHD prophylactic regimens. In MA‐Bu (a) and RIST‐Bu (b) groups, patients receiving ATG prophylaxis (and HSCs from non‐sibling‐matched donors) had a higher incidence of grade 3–4 acute GvHD than their counter partners while there was no such difference in the MA‐TBI group (c). Figure S4. Cumulative incidence of graft‐vs.‐host disease (GvHD) stratified by different conditioning regimens (MA‐Bu vs. MA‐TBI) and GvHD prophylactic regimens. There was no difference between MA‐Bu and MA‐TBI groups regarding the incidence of grade 3–4 acute GvHD (a and b), grade 2–4 (c and d), and all‐grade chronic GvHD (e and f). [file CNR2-5-e1488-s001.pdf]
